# Minimum lethal concentration of sodium hypochlorite for the amphibian pathogen *Batrachochytrium dendrobatidis*

**DOI:** 10.1371/journal.pone.0176439

**Published:** 2017-04-25

**Authors:** Matthew H. Becker, Brian Gratwicke

**Affiliations:** 1 Smithsonian Conservation Biology Institute, National Zoological Park, Washington DC, United States of America; 2 Department of Biology and Chemistry, Liberty University, Lynchburg, Virginia, United States of America; Universiteit Gent, BELGIUM

## Abstract

Sodium hypochlorite (NaOCl) is the active ingredient in household bleach and is commonly used as a disinfectant to clean equipment contaminated by the amphibian pathogen *Batrachochytrium dendrobatidis* (Bd) in lab husbandry and field studies. We conducted a series of replicated exposure trials using a single Global Pandemic Lineage Bd isolate from Panama (JEL 310) and concentrations of NaOCl ranging from 0.006% to 0.6% for exposure times ranging from 30 seconds to 15 minutes to determine the minimum lethal concentration of NaOCl for this isolate of Bd. Sodium hypochlorite completely killed Bd at a concentration of 0.03% during a 15-minute exposure time, while 0.12% NaOCl was effective at all exposure times (30s-15min).

## Introduction

Sodium hypochlorite (NaOCl), the active ingredient in household bleach, is commonly used as a disinfectant to decontaminate equipment and reduce the spread of the amphibian pathogen *Batrachochytrium dendrobatidis* (Bd). Despite other chemicals being recommended for this purpose [1,2], the availability and low cost of bleach has ensured its widespread use [3]. One commonly used field protocol recommends diluting household bleach with 9 parts of water (making a solution of 0.6% NaOCl) to disinfect equipment [4]. This approach has been successfully used to clean enclosures in active Bd experiments [5], and is the recommended concentration for husbandry to manage captive assurance colonies of amphibians in Panama [6]. However, these concentrations seem excessive and projects like the Panama Amphibian Rescue and Conservation Project have been using several cases of bleach per month (R. Ibáñez, pers. comm.).

Two published studies on this topic report different effective concentrations of NaOCl for killing Bd. One study found effective concentrations of 0.2% NaOCl for a 10-minute exposure and 1% NaOCl for a 30-second exposure [2], and a second reported 0.2% NaOCl for a one-minute exposure [1]. However, neither of these studies established a clear minimum lethal concentration. In practical terms, this means that excessive concentrations of bleach as high as one part household bleach to three parts of water are being used in practice [3]. While these concentrations achieve the objective, they are not cost-effective or environmentally-friendly when used at scale and require multiple rinses to avoid any residual exposure to animals. The objective of this study was to determine the minimum lethal concentration of NaOCl for practical purposes of preventing Bd transmission in amphibian husbandry associated with experiments and conservation efforts involving a Panamanian Bd isolate.

## Methods

We used established protocols to determine the minimum lethal concentration of NaOCl [2; 7]. Briefly, we added 150ul of tryptone and 50ul of a 20-μm filtered three-day old Bd culture to each well of a 96-well flat bottom plate. This amounts to approximately one million zoospores of the Global Pandemic Lineage (GPL) JEL 310 (isolated from *Smilisca phaeota*, Fortuna, Panama [8]. The plate was incubated for 72 hours at 23C and then all liquid (200ul) was carefully removed from each well as to not disturb the zoosporangia attached to the bottom of the well. We diluted a 6% stock solution of household bleach (Purebright, KIK international) with Milli-Q ultrapure water to create a serial dilution of 0.006, 0.03, 0.06, 0.12, 0.24, 0.36, 0.48, and 0.6% NaOCl. We added 200ul of bleach dilutions or Milli-Q ultrapure water (positive control) to each well with various exposure times (0.5, 1, 5, and 15 min). Each treatment (concentration x exposure time) was replicated eight times (positive controls were replicated four times). After the appropriate exposure time, we carefully removed all bleach or water from each well, rinsed twice with 1% tryptone, and then added a final 200ul aliquot of 1% tryptone. Bd growth was evaluated by comparing the optical density (at 490nm) of each well at the start of the experiment to the optical density after four days of incubation at 23C [7]. We visibly scanned for new growth every week for four weeks to ensure wells that showed complete inhibition had not become contaminated. The minimum lethal concentration was calculated as the lowest concentration for a specific exposure period in which there was complete inhibition as shown by a 0.0 change in optical density and no visible growth.

## Results

**Fig 1:**
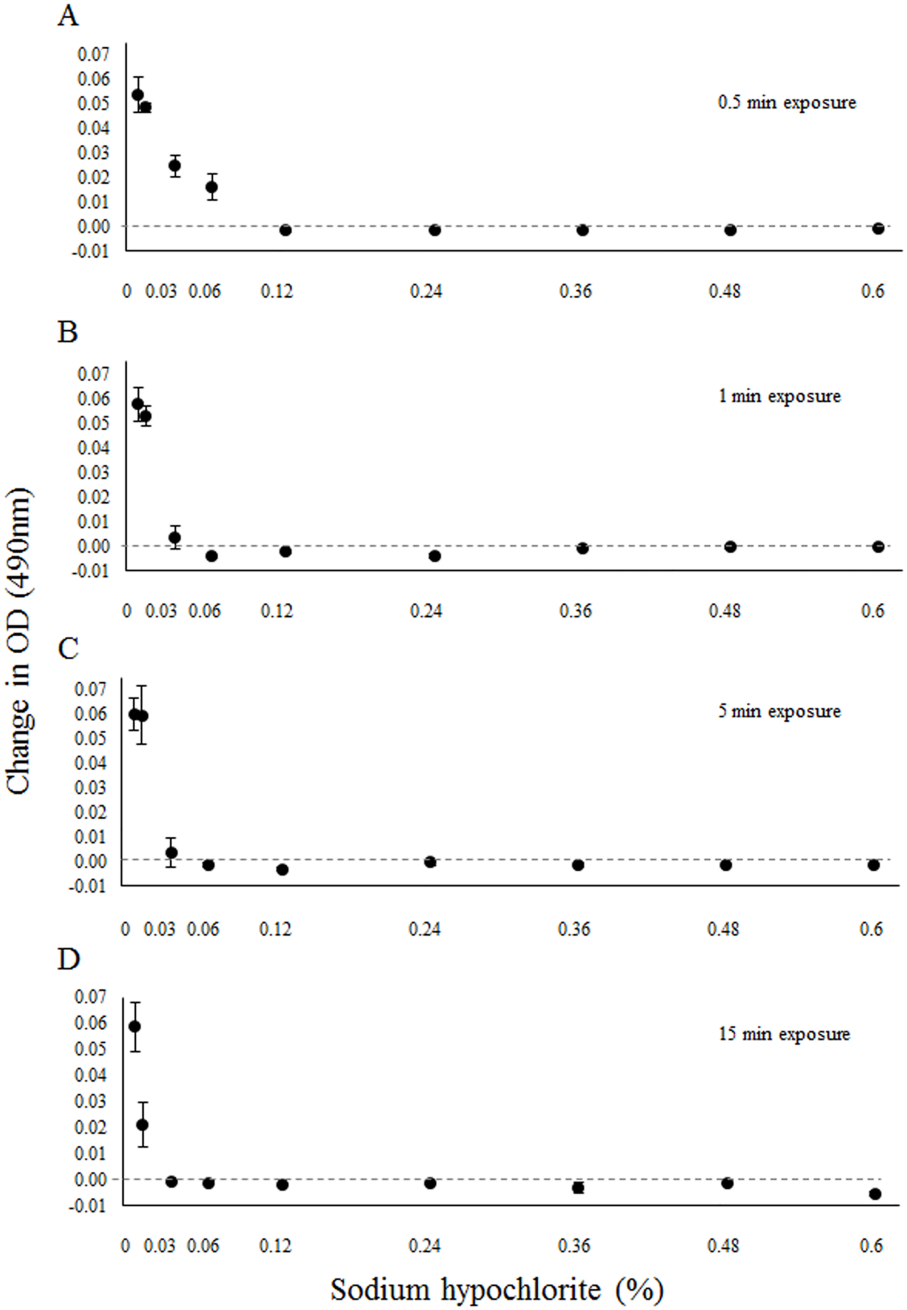
The effect of sodium hypochlorite on the growth of *Batrachochytrium dendrobatidis* (Bd). Change in optical density (OD) of Bd four days after exposure to various concentrations of sodium hypochlorite for (A) 0.5 min, (B) 1 min, (C) 5 min, and (D) 15 min. Error bars represent standard error.

We found the minimum lethal NaOCl concentration for a 30-second exposure was 0.12% (Fig 1A) and that was reduced to a concentration of 0.06% for exposure times of one to five minutes (Fig 1B and 1C). Sodium hypochlorite was effective at killing Bd at concentrations as low as 0.03% if exposure time was increased to 15 minutes (Fig 1D). Supporting data are provided as S1 File.

## Discussion

Two other studies have clearly documented that NaOCl can effectively be used as a disinfectant to prevent the spread of Bd, but both studies were considering a panel of other potential disinfectants and neither sought to establish a clear minimum lethal concentration for NaOCl [1; 2]. In the present study, we only tested a single GPL isolate from Panama, and we do not consider variation in strain susceptibility. NaOCl was highly effective at completely killing Bd at concentrations as low as 0.12% with a 30-second exposure. In practical terms, this means that regular strength household bleach with ranges from 4–6% NaOCl can be diluted with 32–49 parts of water to effectively disinfect pre-cleaned cages and field equipment with exposure times of 30 seconds. It is worth noting here that this method will not destroy Bd DNA which may still contaminate surfaces and remain detectable by PCR. To be effective at destroying DNA, pure bleach 6–12% NaOCl and exposure times of an hour are required [9].

Using higher concentrations than the minimum lethal concentration or longer exposure times are both potential additional precautionary measures because effectiveness may be reduced at lower temperatures, or in the presence of dirt or porous surfaces with poor bleach penetration, or with different untested Bd isolates. However, these results provide us a framework to make decisions informed by a scientifically determined minimum lethal concentration. For those also wishing to also control ranavirus infections 0.18% NaOCL (1 part 6% NaOCl to 32 parts water) was effective at 1 minute exposure time [10], and our results indicate that this concentration and exposure time would be also be highly effective against this Bd GPL isolate.

## Supporting information

S1 File Optical density changes in relation to bleach concentration and exposure time. Excel file containing experimental data with bleach concentration, exposure time, starting and final optical density information used to draw Fig 1.
(XLSX)
